# Transactions of the Medical Society of the State of New York, Vol. II, Part I. 1847

**Published:** 1847-06

**Authors:** 


Art. V.—*Transactions of the Medical Society of the State of New York.* Vol. VII. Part I. 1847.

The first paper in this volume is an address, by Dr. John McCall, on "Mental Manifestation." The brain is shown to be the organ through which the mind manifests itself, and the health or disease, the good or defective organization of which determines the soundness and vigor of the mind's action. A fact illustrative of this now universally received idea, the author gives as occurring under his own observation:

"While surgeon in the United States army, during the last war with England, I had an opportunity of witnessing the effects of compression by my hand on the brain of a soldier, whose skull had been extensively fractured, and a portion thereof, as well as brain, cut away on the upper part of the left hemisphere, by an Indian with his tomahawk. The intellects were scarcely at all impaired; yet on compressing the brain with my fingers, loss of consciousness and insensibility supervened; on removing the pressure, perception and understanding of what was being done around him returned after a short time. Several such cases have been met with and detailed in medical and surgical works. A patient of mine lived two years without knowing the fact. He had been insane, and the last two years of his life were spent in a state of profound idiocy. This was called a disease purely of the mind, and many so regarded it, as the patient was fat and plethoric at the time of death. But on a careful post-mortem inspection of the brain, in the presence of Dr. Brigham and others, it was found extensively diseased. This pathological state accounted most satisfactorily to our minds for the failure of mental manifestations, as stated."

The second paper is by Dr. N. S. Davis, on "Some of the most common diseases of the digestive organs." Extracts from this article will be found in another part of this Journal.

"The Resources of the Medical Profession" is the title of the third article, by Dr. Joseph Bates. The writer gives some melancholy statistics showing the resources of quackery. For example—

"A certain writer remarks that 'Brandreth, with his pills, has risen from a poor man to be a man of extensive fortune. He has now at Sing Sing a three-story factory for grinding his medicines. Aloes are carried into it by the ton, and whole cargoes of the pills are despatched to every part of the Union and down everybody's throat. He has expended $35,000 in a single year for advertising.' He observes that 'Moffat, adding bitters to pills, has run up a fortune of nearly $300,000.' That 'Sherman, once a poor man, by the sale of his lozenges has come to be a buyer of lots and houses by the wholesale.' And that 'Swaim, of Philadelphia, by pouring his panacea into people's stomach, can afford a single pearl head-band for his daughter, worth $20,000.' The writer closes with this remark: 'Your literary man will starve in his garret, while your pillmaker will emerge from his garret into a palace.' A careful investigation of the success of quackery and the encouragement it receives from all classes would lead one to conclude that it must ultimately obtain a conquest, and triumph gloriously over all the resources that the science of medicine can afford to the sufferings of life. We see quackery bolstered up and fostered by the learned and unlearned, the honorable and dishonorable, judges, lawyers, clergymen, and editors of public prints. Here all grades of society meet on one common level, and are all equally duped."

He shows that even Mexico, benighted as she is in many respects, in this is ahead of us:

"Mexico, in her legislation to protect her citizens from the ravages of quackery, is in every particular worthy of imitation. Her 'physicians must be graduated doctors of medicine,' but before they are permitted to practise they must pass a rigid examination. Her apothecaries also by law are subjected to a similar examination. In her legislation she has done all in her power to protect, not her physicians, but her whole commmunity, from the fatal effects of quackery."

The fourth is a paper by Dr. A. H. Stevens on the "diagnosis of nervous diseases." He gives the following case, the like of which must have occurred in the practice of most physicians:

"During the prevalence of spinal irritation, I was asked to see a lady recently from England, by way of Jamaica, whither she had been sent for a supposed consumption, with spinal irritation at the same time. She had been confined to the bed with few exceptions more than two years. Perceiving after a few visits that lively conversation made her forget her ailments, and that the general assemblage of symptoms did not belong to any nosological disease, I obtained from her married sister some matters of private history that led me to be quite sure her case was purely nervous. "My dear doctor, do you know any thing that would cure my poor sister?" "Yes, I do—I think I know—I am sure of it." "What, pray tell me what." "That cat-o'nine-tails hanging over your fireplace!" I will not detain you to describe the scene which followed. About six weeks afterwards I was stopped in Broadway by two highly dressed ladies, one of whom tapped me on the shoulder and introduced my patient to me, saying with a smile in which the latter joined, "that last prescription cured my sister."

